# Tests of Fiber Cement Materials Containing Recycled Cellulose Fibers

**DOI:** 10.3390/ma13122758

**Published:** 2020-06-18

**Authors:** Tomasz Gorzelańczyk, Krzysztof Schabowicz, Mateusz Szymków

**Affiliations:** Faculty of Civil Engineering, Wrocław University of Science and Technology, Wybrzeże Wyspiańskiego 27, 50-370 Wrocław, Poland; krzysztof.schabowicz@pwr.edu.pl (K.S.); mateusz.szymkow@pwr.edu.pl (M.S.)

**Keywords:** fiber cement boards, recycled cellulose fibers, nondestructive testing, acoustic emission, artificial neural networks, SEM

## Abstract

This paper presents the results of investigations into the effect of the use of recycled waste paper cellulose fibers on the properties of fiber cement boards subjected to contamination by moisture. Four series of fiber cement boards were tested. A reference fiber cement board manufactured without the use of recycled cellulose fibers constituted as one of the series. The other three series consisted of boards differing in their recycled cellulose fiber content-ranging from 10% to 50% of the total cellulose fiber content. Specimens of the fiber cement boards were subjected to contamination by moisture by storing them in water for 1–96 h. Subsequently, their basic physical and mechanical parameters, i.e., mass moisture content, absorbability, and modulus of rupture (*MOR*), were tested. Then, the specimens were investigated by means of acoustic emission during three-point bending. Artificial neural networks were employed to analyze the acoustic emission test results. The tests clearly showed the amount of recycled waste paper cellulose fibers and the length of storage in water to have an adverse effect on the boards, contributing to their degradation. This was reflected in the decrease of the acoustic emission (AE) events count recognized by the artificial neural networks, accompanying the rupture of fibers during the three-point bending of the specimens. In order to gain a more detailed insight into the changes taking place in the structure of the tested fiber cement boards, optical examinations were carried out by means of a scanning electron microscope. Interesting findings crucial for building practice were noted.

## 1. Introduction

In building practice, it is often observed that under the impact of weather conditions, e.g., temperature changes and excess moisture, the physical and mechanical properties of exterior wall cladding deteriorate and eventually, the latter, is damaged. Currently, fiber cement boards are very often used for cladding the façades of buildings. Such boards are a building product, which has been used in construction since the beginning of the last century. The Czech engineer Ludwik Hatschek developed and patented the technology of producing this composite material. The first boards contained asbestos fibers. After asbestos had been found to be carcinogenic, it was replaced with mainly cellulose fibers and synthetic fibers [1]. The currently manufactured fiber cement boards consist of cement, cellulose fibers, synthetic fibers, as well as various innovative additives and admixtures and are a completely different building product. Fiber cement boards’ other constituents and fillers are limestone powder, mica, perlite, kaolin, microspheres, and recycled materials [2,3], whereby such boards are innovative products consistent with the principles of sustainable development [4]. Fiber cement boards are used in construction mainly as ventilated façade system cladding [5]. In the course of their service life, fiber cement boards are exposed to variable environmental factors, chemical aggression (acid rains), physical aggression (ultraviolet radiation), and high temperatures (e.g., during a fire) [6,7,8,9]. Considering the requirements of sustainable development and the search for innovative products, various additives and recycled materials, e.g., recycled waste paper cellulose, were used for the production of fiber cement boards [2,10,11]. The aim is, on the one hand, to create an environment-friendly product and, on the other hand, to reduce the production costs. However, this raises the question concerning the physical and mechanical properties of such boards, especially their durability. The present paper addresses this question, presenting the results of tests of the selected physical properties (absorbability and mass moisture content) and mechanical properties (the modulus of rupture (*MOR*)) of selected commercially available fiber cement boards manufactured using waste paper and without using waste paper. The mass moisture content and the modulus of rupture (*MOR*) were tested since, according to standard [12], these are the principal parameters indicative of the fiber cement board’s resistance to weather conditions and its durability. However, these are basic tests, which do not give an insight into the destructive changes taking place in the structure of the tested boards under the action of the above factors. In the authors’ opinion, such changes certainly take place and can affect the further service life of the boards. In order to prove this thesis, specimens of fiber cement boards characterized by various degrees of moisture buildup were investigated through acoustic emission (AE) during three-point bending. The results of the investigations were analyzed using artificial intelligence (AI), namely, artificial neural networks. Previously, the authors found that an assessment of the effect of many service factors contributing to structural damage to the tested fiber cement boards, based solely on the modulus of rupture (*MOR*), was inadequate [7,8,9,13,14]. This time, using the acoustic emission method to examine the degeneration of the tested samples, the authors were able to describe the destructive changes in the structure of the tested boards on the basis of not only the mechanical parameters, but also the acoustic phenomena occurring in such boards. The recorded acoustic emission (AE) signals were subjected to an analysis, and on its basis reference, acoustic spectrum patterns accompanying respectively the fracture of the cement matrix and the rupture of the fibers during bending were determined. Next, the reference patterns were recognized in the AE records by means of artificial neural networks. In order to verify the results and to gain a better insight into the changes taking place in the structure of the boards, the latter was optically examined using a scanning electron microscope (SEM).

## 2. Survey of Literature

So far, the effect of service factors [15,16,17] and high temperatures on fiber cement boards has been studied through their physicomechanical properties, mainly their modulus of rupture (*MOR*). There have been few publications dealing with the investigation of boards containing recycled waste paper cellulose fibers [2,10,11]. So far, only a few investigations of fiber cement boards have been conducted using nondestructive methods, including the acoustic emission method [18]. These are mainly studies carried out by the present authors.

The impact of high temperatures on fiber cement boards was examined, but only through *MOR*, in [11]. The effect of high temperatures on extruded composites was studied on the basis of solely the composite’s mechanical properties by Li et al. [19]. The use of nondestructive methods to assess the effects of high temperatures, fire, and freeze/thaw cycling on the degree of degradation of fiber cement boards on the basis of not only physicomechanical parameters was presented in [7,8,9,13]. Other reported nondestructive investigations of fiber cement boards concern mainly the detection of imperfections arising during production [20]. Stark et al. in [21] described a method of detecting delaminations in composite elements by means of a moving ultrasonic probe. An ultrasonic device and a method of detecting delaminations in fiber cement boards were described by Dębowski et al. in [22]. Drelich et al. [23] and Schabowicz et al. [24] presented the possibility of using Lamb waves in a contactless ultrasonic scanner for detecting defects in fiber cement boards already at the production stage. In the literature on the subject, there is still little information on the application of other nondestructive methods to test fiber cement boards. Preliminary studies by Chady et al. [25,26] showed the terahertz (T-ray) imaging method to be suitable for testing fiber cement boards. Terahertz signals are similar to the ones obtained using the ultrasonic method, but their interpretation is more complicated. Ranachowski and Schabowicz et al. [27,28] used the microtomography method to indicate delaminations and regions of low density in fiber cement boards. This method can be a useful tool for testing the structure of fiber cement boards in which defects can arise due to manufacturing faults. However, the method can be used only for small boards. As mentioned earlier, so far few AE investigations of fiber cement boards have been reported. It is worth noting that the pilot acoustic emission study of extruded fiber cement boards, including the ones exposed to a temperature of 230 °C for 2 h, aimed at determining the effect of cellulose fibers on the strength of the boards and attempting to distinguish between the AE events emitted by the fibers and the cement matrix, respectively, carried out by Ranachowski et al. The authors confirmed the suitability of the acoustic emission method for testing fiber cement boards. Schabowicz et al. [7,8] and Gorzelańczyk et al. [9,13] proposed to use the AE method to examine the effect of fire and high temperatures and that of freeze/thaw cycling on fiber cement boards. It should be noted that the effect of high temperatures on concrete and the relevant dependences, determined using the acoustic emission method, are widely described in the literature, e.g., in papers by Ranachowski [29,30] and Ranachowski et al. [29,31]. A very large amount of data, which need to be properly analyzed and interpreted, are recorded during acoustic emission measurements. For this purpose, it is useful to combine the acoustic emission method with artificial intelligence, including artificial neural networks (ANNs). In the literature, ANNs are used to analyze and recognize signals acquired during the degradation of various materials [32]. Schabowicz [33] used ANNs to analyze the results of nondestructive tests of concrete. Łazarska et al. [34] and Woźniak et al. [35] successfully used the acoustic emission method and artificial neural networks in steel testing. Rucka et al. [36,37] successfully used the acoustic emission method to investigate damage to concrete structures. Furthermore, the present authors in [7,8,9,13] successfully used ANNs to analyze the results of investigations of fiber cement boards subjected to the action of fire and high temperatures and to freeze/thaw cycling.

Considering the above, the authors concluded that the acoustic emission method combined with artificial neural networks would be proper for assessing the destructive changes taking place in the structure of fiber cement boards manufactured using recycled waste paper cellulose and damp to different degrees.

## 3. Tests of Principle Physical and Strength Properties

The subject of this study were fiber cement boards. The basic composition of the raw materials used in the manufacture of fiber cement boards is given in Table 1. Different products can be manufactured from the same components depending on their mutual proportions.

One should note that recycled waste paper cellulose was shorter and had lower mechanical parameters by 30–50% than normal cellulose and must be subjected also to special impregnation [1,2,4]. This treatment increases the biological stability of the cellulose fibers. The best results are achieved when the cellulose fibers are treated with didecyldimethylammonium chloride (DDAC) or didecyldimethylammonium bromide (DDAB). Thanks to these substances combined with a low copper content, the product is characterized by very high biological stability at no high expenditure of treatment energy or fiber length loss [1].

Table 2 shows the requirements concerning selected raw materials (cellulose fibers, cement, and typical additives) used for the production of fiber cement boards.

Considering the above, the authors carried out tests on four series of fiber cement boards denoted with letters from A to D, specified in Table 3, making the following assumptions. Board A was adopted as the reference board manufactured in accordance with the composition shown in Table 1, with no addition of recycled cellulose fibers. Whereas boards B, C, and D contained recycled cellulose fibers amounting to 10%, 25%, and 50% of the total cellulose fibers content (by weight).

The testing regime and the testing methods used are briefly presented below.

In regards to the physical properties, absorbability *n_w_* and mass moisture content *w_m_* were determined. Absorbability *n_w_* was determined for boards stored in water for 1, 2, 3, 24, 48, 72, and 96 h, from the relation:

(1) $$n_{w}=\frac{m_{n}-m_{s}}{m_{s}}\;\left[\%\right]$$

where:

*m_n_*—the mass of the water saturated sample [g];
*m_s_*—the mass of the sample dried at 105 °C [g].

Using the drying–weighing method, the mass moisture content *w_m_* was determined for board samples in dry-air condition (prior to the test stored in laboratory conditions) from the relation

(2) $$w_{m}=\frac{m_{w}-m_{s}}{m_{s}}\;\left[\%\right]$$

where:

*m_w_*—the mass of the sample with the current moisture content [g];
*m_s_*—the mass of the sample dried at 105 °C [g].

The mechanical parameter, i.e., flexural strength (three-point bending, the arithmetic mean for two bending directions), was determined for air-dry boards and for boards stored in water for 1, 2, 3, 24, 48, 72, and 96 h, using 250 × 250 mm square specimens in accordance with [12]. *MOR* [MPa] was calculated for each direction from the formula

(3) $$MOR=\frac{3Fl_{s}}{2be^{2}}$$

where:

F—the rupture force [N];
$l_{s}$—the spacing of the supports [mm];
b—the width of the tested specimen [mm];
e—the tested specimen’s mean thickness measured in four places (in the middle of each of the sides) [mm].

**Figure 1:**
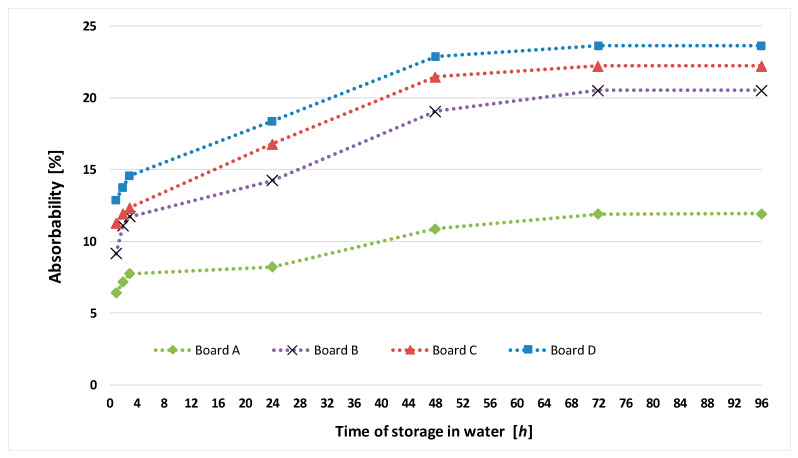
Increase in absorbability *n*_w_ over storage time in water for tested boards.

The averaged (for ten specimens of each type of fiber cement board) test results for absorbability *n_w_* and mass moisture *w_m_* are presented in Table 4. Figure 1 shows the increase in absorbability *n_w_* of the tested boards over storage time in water.

**Figure 2:**
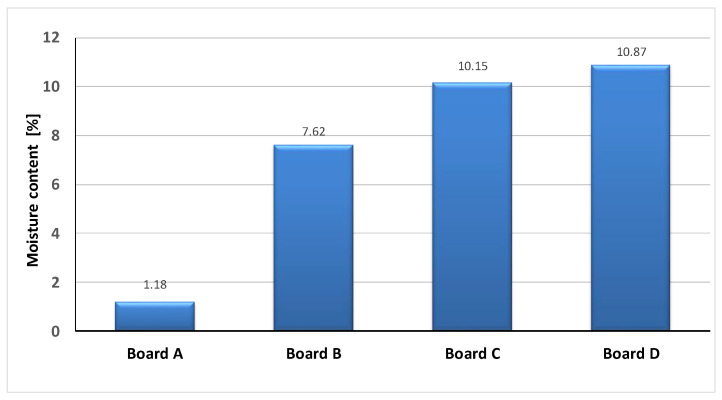
Mass moisture content in tested boards.

The determined values of moisture content *w_m_* for all the tested boards are shown in Figure 2.

The averaged (for ten samples of each type of board) *MOR* values of the fiber cement boards tested in accordance with [12] are presented in Table 5.

**Figure 3:**
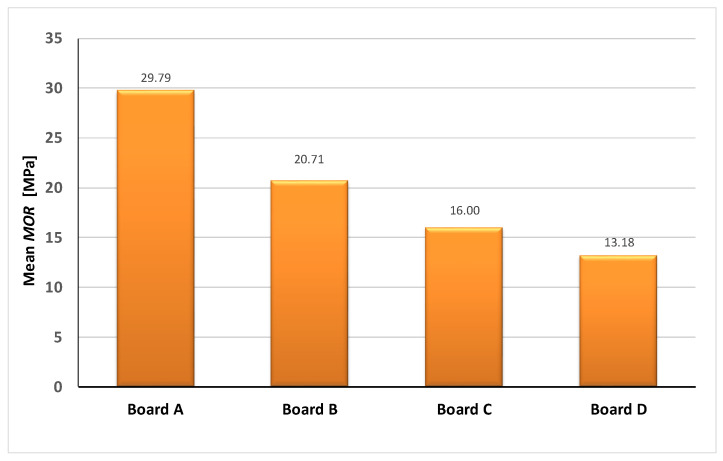
Mean modulus of rupture (*MOR*) of boards tested in air-dry condition.

Figure 3 shows bending test results for the boards in air-dry condition at the instant of testing.

**Figure 4:**
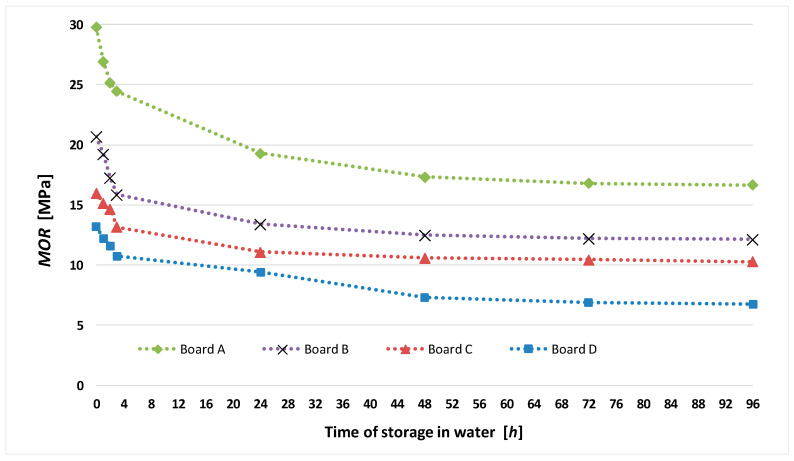
*MOR* of tested boards versus time of their storage in water.

It appears from Table 5 and Figure 3 that air-dry board A is characterized by the highest modulus of rupture, whereas board D (with the 50% recycled cellulose content) has the lowest strength. Moreover, analyzing Table 5, one should note that board D is characterized by practically the largest decrease in strength—from 13.18 to 6.76 MPa—due to moisture buildup. The best modulus of rupture parameters under storage in water characterized board A containing no recycled materials—the decrease in strength was the smallest in this case. The above findings are best illustrated in Figure 4, which shows the decrease in the modulus of rupture of the fiber cement boards over time of their storage in water.

Analyzing the graphs in Figure 4, one should note that as the recycled cellulose content in the boards increases, there is a tendency towards a larger decrease in *MOR* due to moisture buildup in comparison with the boards with the same composition, but manufactured without using recycled waste paper cellulose.

## 4. Testing by Means of Acoustic Emission and Artificial Neural Networks

**Figure 5:**
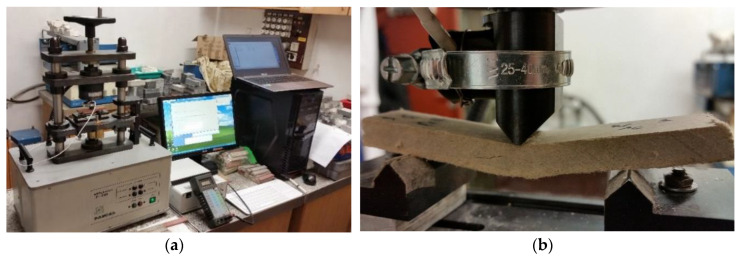
Stand for measuring acoustic emission during a three-point bending test: (**a**) instrumentation setup, (**b**) fiber cement board specimen during testing.

In order to verify the above findings, the fiber cement boards were tested by means of acoustic emission and artificial neural networks. A broadband sensor with a frequency response of 5 kHz–500 kHz and an AE signal analyzer were used for AE measurements. Figure 5 shows the stand for measuring acoustic emission during the bending test conducted on the fiber cement boards.

**Figure 6:**
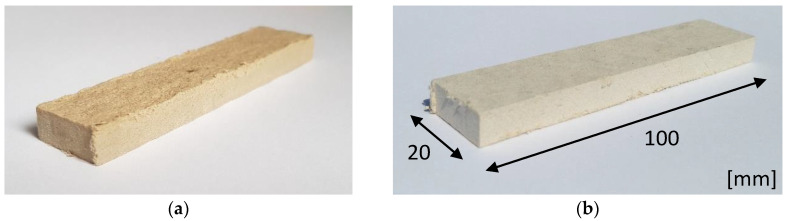
Exemplary specimens of fiber cement boards belonging to (**a**) series C air-dry and (**b**) series C stored in water for 24 h.

Prior to the tests, ten 20 × 100 mm fiber board specimens of each of the series (A, B, C, and D), air-dry and stored in water for 1, 2, and 24 h, were prepared. Exemplary specimens of series A and B boards are shown in Figure 6.

**Figure 7:**
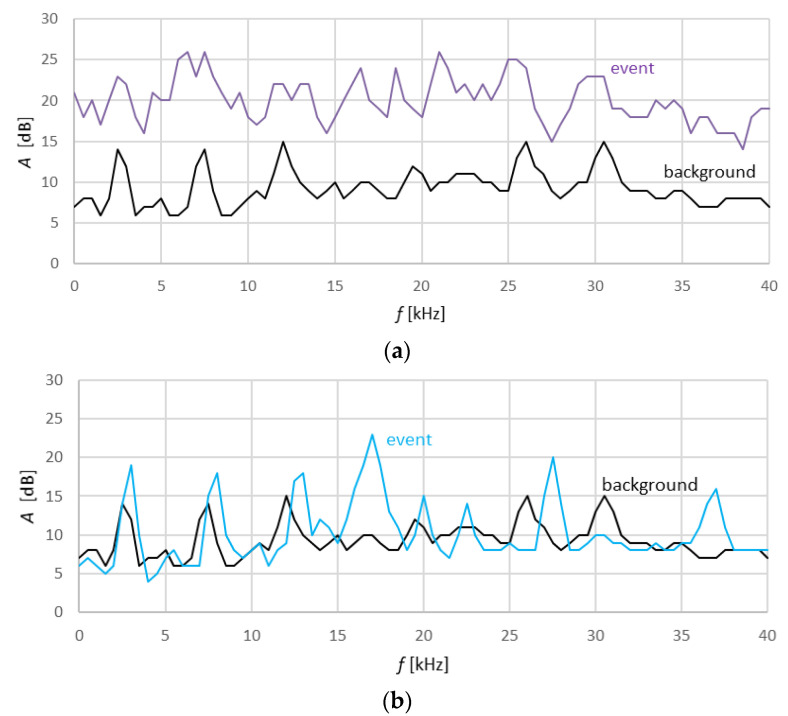
Exemplary record of spectral characteristic of (**a**) high-energy and (**b**) low-energy event and background noise.

The specimens prepared in this way were placed in a strength testing machine and subjected to three-point bending during which rupture force *F* and acoustic emission signal traces were recorded. The analysis of the test results covered the trace of flexural stress *σ*_m_, *MOR*, and the limit of proportionality (LOP), which is the limit of Hooke’s law. The analysis of this parameter is important from the point of view of the elastic properties of the material and their changes under the influence of the use of recycled fibers and under the influence of storage in water. The AE signals were analyzed on the basis of the recorded descriptors, such as acoustic emission events count ∑*N_ev_* and AE events rate *N_ev_*, and on the basis of the AE signal frequency distribution. Figure 7 shows an exemplary record of a spectral characteristic of a high- and low-energy event, and an approximate characteristic of the background noise registered during the bending test.

**Figure 8:**
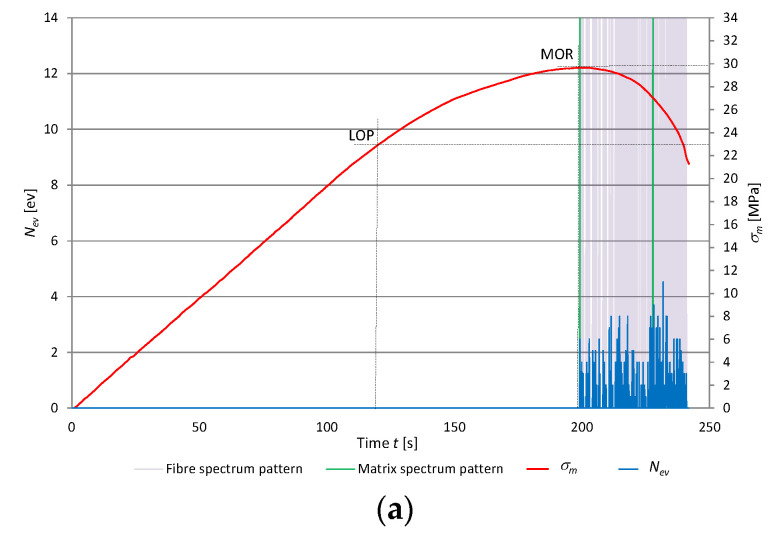

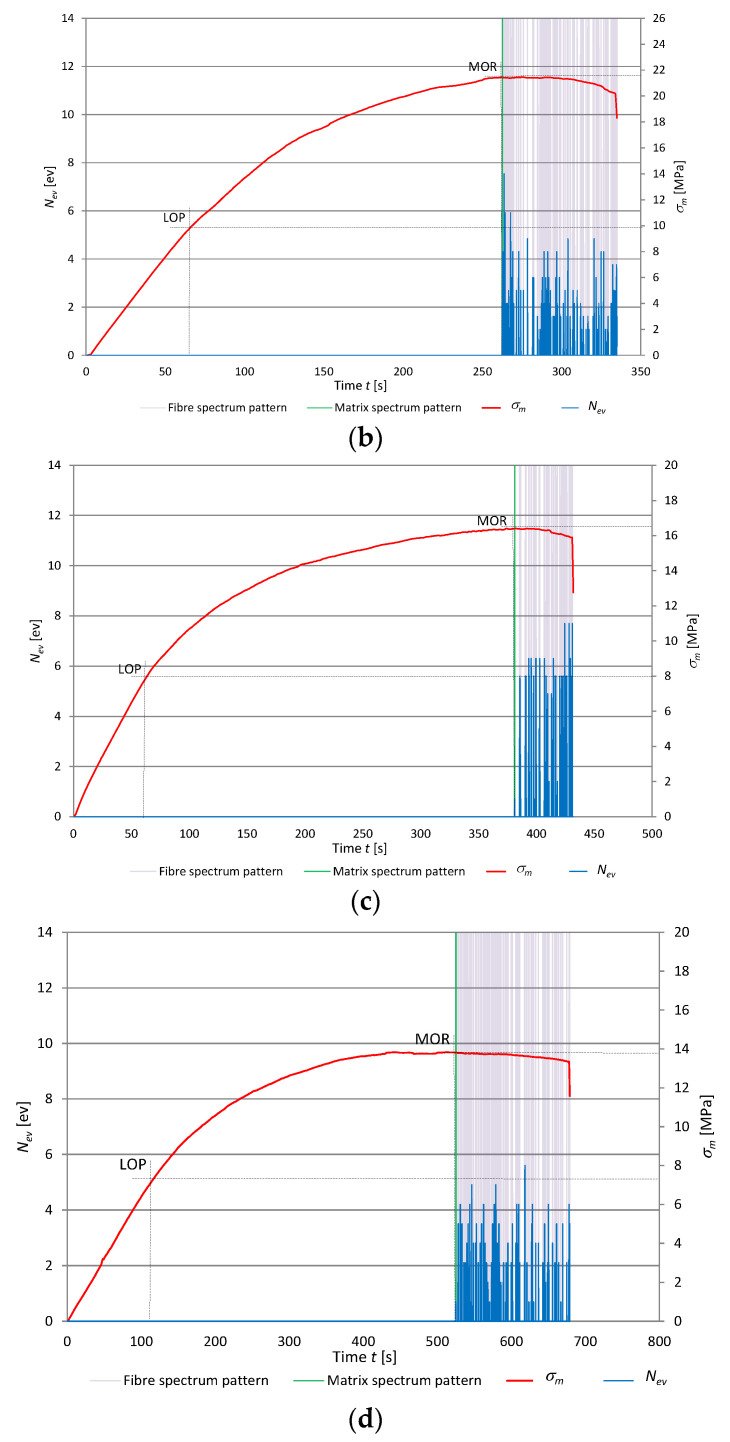
Events rate *N*_ev_ and flexural stress *σ_m_* versus time for air-dry fiber cement boards, with marked identified reference spectral patterns: (**a**) series A, (**b**) series B, (**c**) series C, (**d**) series D.

High-energy AE events are most often registered by the sensor and they can be quite easily recognized. Whereas low-energy events can be drowned out by the background noise generated by the microtester, and thus are difficult to recognize. A spectral analysis of the acoustic spectrum can reveal events characterized by different acoustic activity, including the low-energy ones accompanying the rupture of fibers. In the tests, the spectral density function covered the frequency range of 1–40 kHz in 80 intervals at every 0.5 kHz, representing the sum of its frequency components. In this way, the signal was presented in the frequency domain. Artificial neural networks were used to recognize and separate the signals generated by the fracture of the cement matrix from the signals generated by the rupture of the fibers contained in this matrix. In the course of three-point bending, a very large number of AE events are registered. They originate from various processes and can occur simultaneously. The artificial neural network recognition of the acoustic emission (AE) signals registered during bending was carried out in three steps. First, input data—reference spectral patterns experimentally assigned to the signals accompanying respectively the rupture of fibers and the fracture of the matrix and originating from background noise—were acquired. For the needs of the neural classification algorithm, the reference spectral patterns were converted through binary encoding into appropriate zero-one sets. Unidirectional multilayer back propagation artificial neural networks were employed to recognize the patterns. Then, the ANN training patterns were implemented in appropriate combinations and numbers of iterations. The obtained ANN training and testing results, i.e., the ANN’s structure and weighs, were saved and constituted the trained artificial neural network to be used for AE signals recognition. Finally, the AE signals registered during bending were filtered and the spectral patterns accompanying the rupture of fibers and the fracture of the cement matrix were assigned. The method which uses artificial neural networks to recognize registered AE signals is described in detail in [7,8,9,15,34,35,38]. The registered events are distinguished not so much by their energy as by their spectral characteristic. Therefore, a spectral analysis was carried out to recognize the spectral characteristics and respectively to assign them to the background noise, the rupture of fibers, and the fracture of the cement matrix. The way in which the reference acoustic spectra respectively accompanying the rupture of the fibers and the fracture of the cement matrix are singled out is described in [8,9]. The fiber content in fiber cement boards and the parameters of the fibers determine the properties of the composite, especially its modulus of rupture (*MOR*). Hence, an analysis of the AE signals attributed to the rupture of the fibers during bending is key for the evaluation of the properties of boards containing recycled cellulose fibers. Figure 8 shows diagrams of flexural stress σ_m_ and AE events rate *N_ev_* and their assignment to the reference fiber rupture and matrix fracture patterns, respectively, during the three-point bending test for the selected specimens of the air-dry fiber cement boards of series A, B, C, and D.

It appears from the diagrams shown in Figure 8 that the boards containing a larger amount of recycled cellulose fibers are characterized not only by a lower *MOR*, but also by a lower number of registered events originating from the rupture of fibers. Moreover, the loss of elasticity by the board and its considerable deformation during the bending test are important, which increase with the recycled cellulose content in the board composition. The considerable deformation characteristic of the boards with a higher recycled cellulose fibers content (boards C and D) increases in the course of bending. This means that the fibers are not ruptured but pulled out of the cement matrix or stretched.

**Figure 9:**
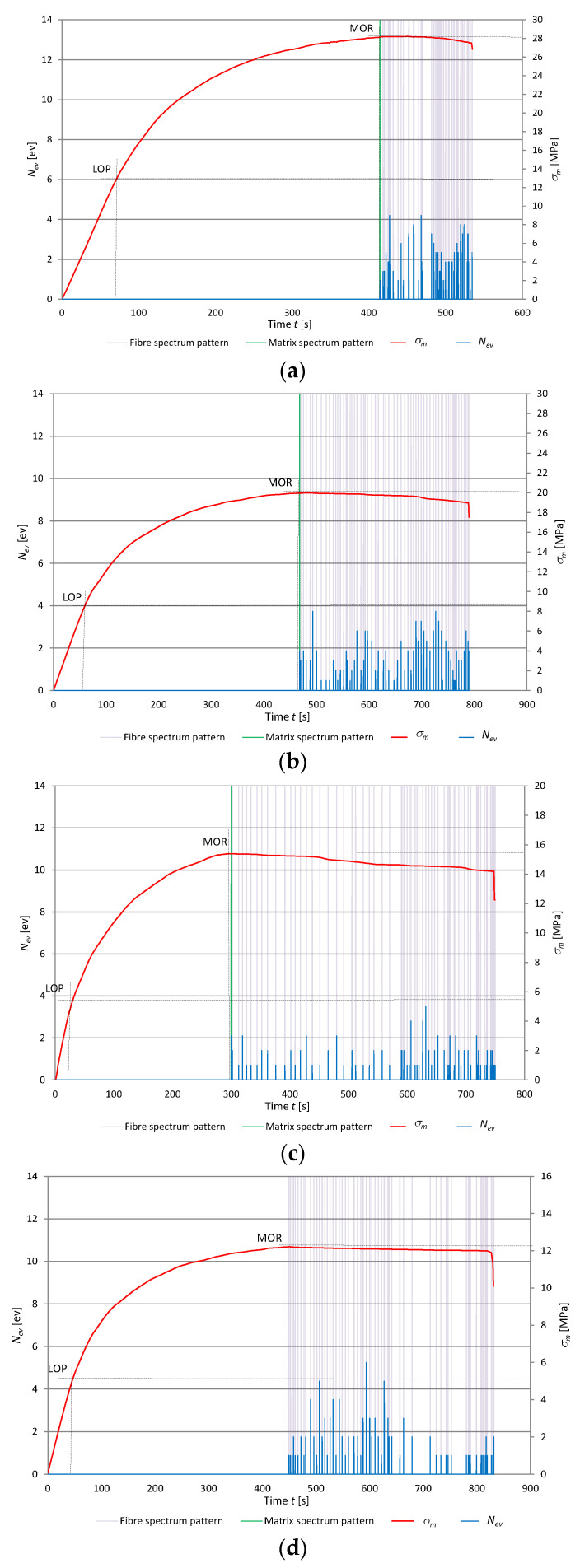
Events rate *N*_ev_ and flexural stress *σ_m_* versus time for fiber cement boards stored in water for 1 h, with marked reference spectral patterns: (**a**) series A, (**b**) series B, (**c**) series C, (**d**) series D.

**Figure 10:**
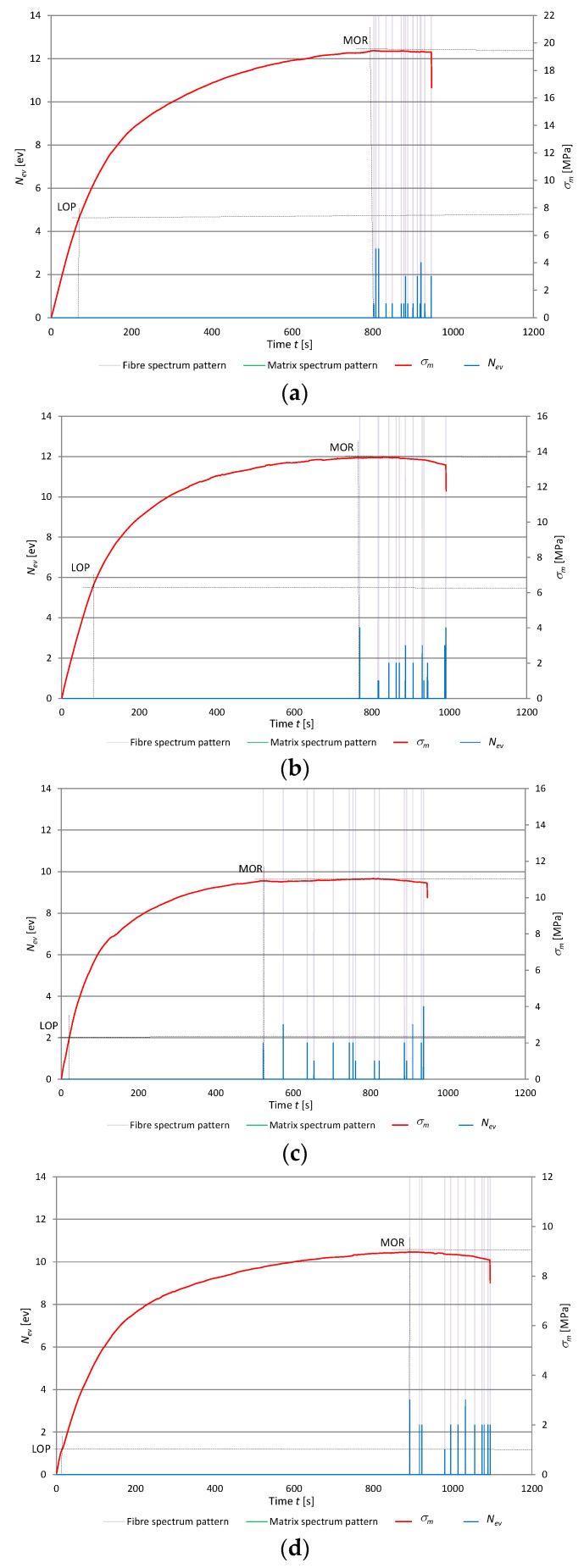
Events rate *N*_ev_ and flexural stress *σ_m_* versus time for fiber cement boards stored in water for 24 h, with marked identified reference spectral patterns: (**a**) series A, (**b**) series B, (**c**) series C, (**d**) series D.

Figure 9 and Figure 10 show exemplary traces of AE events and their recognition by ANNs for boards A, B, C, and D stored in water for respectively 1 h and 24 h. Due to the increased absorbability characteristic of fiber cement boards containing recycled cellulose fibers, the bending test time increases and so does the permanent deformation measured before the failure of the board. The diagrams also show that that storage in water results in lower acoustic activity during bending, i.e., the count of the registered AE events decreases. This decrease is especially marked for the boards containing recycled cellulose fibers. As the recycled cellulose fiber content in the boards increases, the count of registered AE events decreases. During the bending test, recycled cellulose fibers are characterized by low acoustic activity which decays as the time of storage of the boards in water is extended.

One can conclude from the results presented in the above figures that the registered AE events accompanying the rupture of fibers can provide an estimate of the number of fibers contained in the fiber cement boards. However, one should note that recycled cellulose fibers show low acoustic activity during the bending test, as indicated by the smaller number of registered AE events in the boards containing recycled cellulose fibers in comparison with the reference board (series A). In the case of board series C and D, storage in water significantly lowers not only their *MOR*, but also their acoustic activity originating from the rupture of fibers. This is due to the weakening of the fibers and the loss of their adhesion to the cement matrix caused by moisture. A change in the proportionality limit (LOP) value indicates changes in the elastic properties of the panel, including changes in the board structure that have occurred as a result of the addition of recycled fibers. Boards with a higher proportion of cellulose fibers become less elastic as shown in the charts and, in addition, under the effect of storage in water, the elasticity of the plates practically disappears. In boards stored in water for more than one hour for series C and D, the elastic properties of panels are observed only in the initial bending phase. It should also be noted that preliminary studies of fiber cement boards stored in water for longer than 24 h showed a fall in acoustic activity to below the threshold of registrability during the bending test.

**Figure 11:**
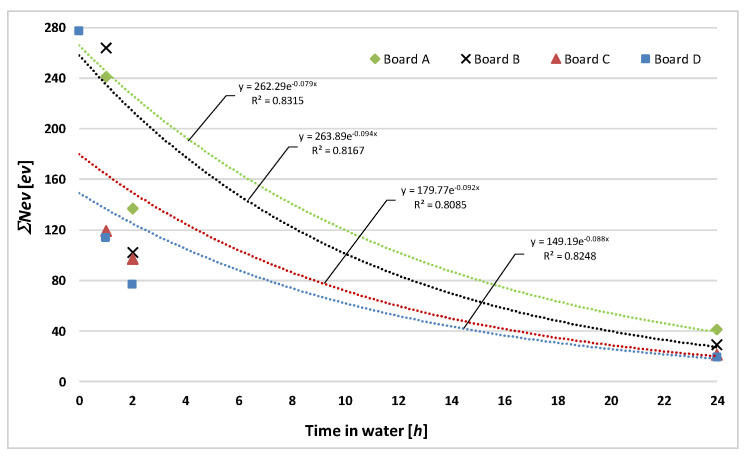
Count of acoustic emission (AE) events recognized as accompanying rupture of fibers for fiber cement boards of series A, B, C, and D versus time of storage in water.

Figure 11 shows a diagram of the count of AE events recognized as accompanying the rupture of fibers for fiber cement boards of series A, B, C, and D, respectively, versus a time of up to 24 h of storage in water.

Table 6 contains the averaged count of the registered AE events attributed to the rupture of fibers.

It appears from the diagrams shown in Figure 11 and the data contained in Table 6 that already after one hour of storing the boards in water a considerable drop in AE events recognized as accompanying the rupture of fibers was recorded for all the tested fiber cement boards. In the case of the boards (series C, D) with a higher recycled cellulose fiber content (25%, 50%), the count of registered AE events was much lower (by about 50%) in comparison with the air-dry boards.

After 24 h of storage in water, the registered AE events were at a similar very low level for all the series of the tested boards. It is worth noting that as the percentage of recycled cellulose in the boards increases, the count of registered AE events tends to be lower, which is due to the lower quality of the fibers and their weaker adhesion to the cement matrix. The investigations showed that the tendency for *MOR* to decrease with increasing time of storage in water (Figure 4) is correlated with the decrease in AE events recognized as accompanying the rupture of fibers (Figure 11), with only up to 24 h of storage in water. After this time, the acoustic activity of the fibers decays and is unmeasurable during the three-point bending test. This means that the acoustic emission tests have confirmed the earlier findings that as the recycled cellulose content in fiber cement boards increases, a tendency towards greater decline in the modulus of rupture of the boards due to moisture buildup, in comparison with fiber cement boards with the same composition but manufactured without the use of recycled cellulose, is observed.

## 5. Scanning Electron Microscope (SEM) Examinations

**Figure 12:**
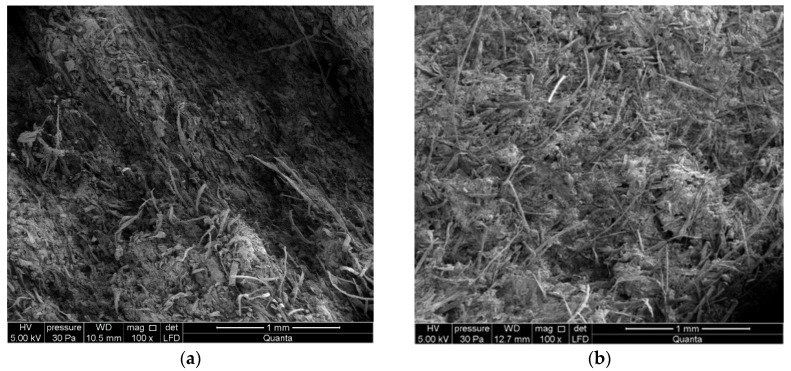
SEM images (magnification 100×) for (**a**) board A and (**b**) board D.

In order to get a more detailed insight into the changes taking place in the structure of the tested fiber cement boards subjected to contamination by moisture, optical examinations were carried out using a high-resolution environmental scanning electron microscope Quanta 250 FEG, FEI with an EDS analyzer. Figure 12 shows exemplary images obtained by means of the microscope for tested fiber cement board A, which did not contain recycled cellulose, and for board D, containing recycled cellulose.

During microscopic observations, the structure was determined to be fine porous with pore sizes of up to 50 μm. Caverns and gouges were visible at the breakthrough site after tearing up to 500 µm wide fibers. Cellulose fibers are clearly visible. A thin layer of cement matrix and hydration products covers the surface of the fibers. Numerous fibers on the fractured surface of board D (with a 50% recycled cellulose content) are clearly visible in the images. Unlike board D, board A is characterized by a more compact structure and a smaller number of fibers, as confirmed by the different density of the two boards and by a considerably higher strength of board A.

**Figure 13:**
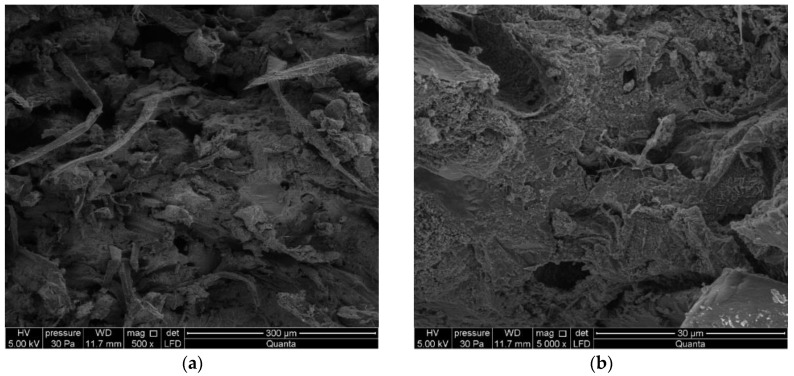
SEM images under magnification 500× (**a**) and 5000× (**b**) for board D after 24 h of storage in water.

Figure 13 shows images of the fractured surface of board D stored in water for 24 h and then subjected to three-point bending.

Numerous bugholes and grooves left by fibers pulled out during the bending test are visible in the images shown in Figure 13. As a result of storage in water, the fibers lost their adhesion to the cement matrix and were unable to carry the stresses generated during bending. Most of the fibers were not ruptured but were pulled out of the bugholes without generating any registrable AE events. It can be concluded from the images shown in Figure 13 that the single events registered during the bending test for the fiber cement boards stored in water for over 24 h originated from the few fibers undergoing rupture, which still did not lose their adhesion to the cement matrix.

## 6. Conclusions

The investigations of the fiber cement boards containing recycled waste paper cellulose fibers showed significant differences in the tested parameters. The following conclusions can be drawn from the test results:

- The recycled cellulose fibers contained in the boards lower their modulus of rupture (*MOR*). The higher the percentage of recycled fibers in the board, the lower the *MOR*;
- Recycled cellulose fibers were found to adversely affect the fiber cement boards by increasing their absorbability. As the percentage of recycled fibers in the boards increased, so did their mass moisture content. As the time of storage of the boards in water increased, the modulus of rupture was found to decline evenly in all the tested fiber cement boards. In the case of the boards with a 50% recycled cellulose fiber content, the decrease in *MOR* due to moisture buildup for 96 h reached 50%, while for the reference board (series A) it amounted to 45%;
- The acoustic emission testing combined with artificial neural networks confirmed (on the basis of the count of recognized AE events accompanying the rupture of fibers) the significant effect of the recycled cellulose fibers on the properties of the fiber cement boards, reflected in the fall in the number or AE events registered during the bending test;
- Already, the storage of the boards in water for 1 h resulted in a significant decrease in the number of AE events recognized as accompanying the rupture of fibers. In the case of the boards containing more than 25% of recycled cellulose fibers, the fall in the number of AE events originating from the rupture of fibers exceeded 50%;
- Recycled cellulose fibers are more susceptible to moisture buildup, which results in the loss of the fiber cement boards’ strength and at the same time in the lowering of their acoustic activity during bending;
- The SEM examinations showed that in the boards stored in water most of the fibers had not ruptured during bending, but had been pulled out of the bugholes in the cement matrix without generating registrable AE events.

**Figure 14:**
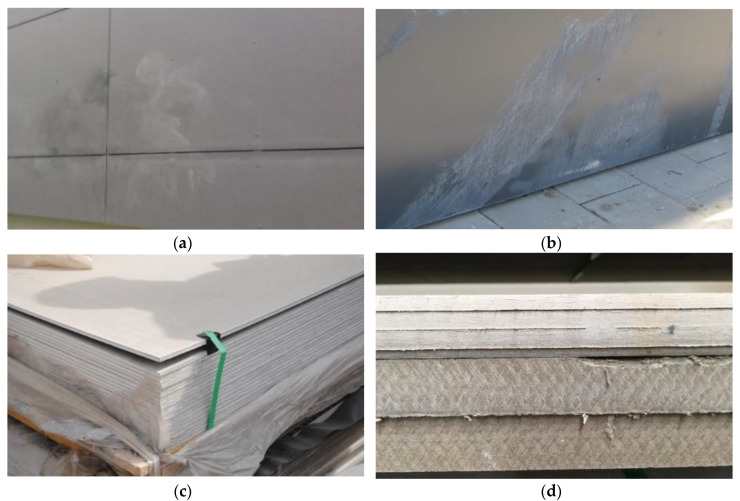
Damaged building façades made of fiber cement boards (**a**,**b**) and warped stock boards containing recycled materials (**c**,**d**).

Moreover, in the authors’ opinion, it is worth noting that the strength parameters of the fiber cement boards, which in a relatively short time became considerably damp, deteriorated and visible deformations appeared on the surface of the boards, causing paint coat peeling and warping. Figure 14 shows examples of damaged building façades made of fiber cement boards containing recycled waste paper materials (having the composition similar to that of board D). The figure also shows boards which warped before their installation as façade cladding.

Based on the experimental results, the authors suggest using recycled cellulose fibers of an amount of less than 5–10% of the total cellulose fiber weight content to manufacture fiber cement boards to be used as ventilated façade system cladding. Currently, there are no effective methods of protecting recycled cellulose fibers against the action of moisture. Therefore, a higher content of such fibers is inadvisable, as it will significantly adversely affect the strength of the manufactured boards. This is a major consideration in the case of boards intended for exterior application, which will be exposed to variable weather conditions. Whereas in the case of fiber cement boards intended for interior application, in the authors’ opinion, a much higher recycled cellulose content can be used since such boards do not have to meet overly rigorous strength requirements.

The above findings are of crucial importance for building practice since fiber cement boards, which contain recycled materials, are characterized by worse parameters, such as absorbability, mass moisture content, and modulus of rupture. This means lower resistance to weather conditions, and thus a shorter service life of façades made of such boards. In the authors’ opinion, fiber cement boards containing waste paper cellulose fibers are an innovative product consistent with the principles of sustainable development, which is excellent for interior applications. In regards to the use of recycled cellulose fibers in the manufacture of exterior (façade) cladding panels, additional treatments need to be applied at the production stage to protect the fibers against excessive water absorption, and then rigorous tests must carried out to confirm the effectiveness of the treatments.

## Figures and Tables

**Table 1:** Exemplary raw materials composition of fiber cement boards [36].

Raw Material	Approximate Percentage
Cement	~60 [%]
Cellulose (dry)	~8 [%]
PVA	~2 [%]
Kaolin or lime	~30 [%]
Total	~100 [%]
Additives and admixtures	
Hyperplasticizer	~0.1 l/t *)
Didecyldimethylammonium (DDAC) or bromide chloride (DDAB)	~0.1 l/t *)
Pearlite	~1 kg/t *)
Mica	~1 kg/t *)
Microsphere	~1 kg/t *)
Antifoaming agent	~0.26 l/t *)

*) liters/ton = liters per ton of ready product.

**Table 2:** Requirements for selected raw materials used in production of fiber cement boards [36].

Raw Material	Requirements				
Cellulose	Pulp				
	Pulping Time	[min]	0	40	70
	Schopper Riegler degrees	[°SR]	12.5	23.9	50.0
	Apparent specific weight	[kg/m^3^]	640	852	905
	Tension coefficient	[Nm/g]	49.9	106.5	112.1
	Rupture coefficient	[kPa/m^2^/g]	3.0	7.4	7.4
	Flexibility	[%]	2.0	3.1	2.9
	Tear coefficient	[mN m^2^/g]	23.7	20.1	19.7
	Kappa number		25 ± 3		
	Dryness	[%]	80		
	Min. fiber length	[mm]	1.6–2.7		
Cement	Portland Cement CEM I 42.5 R Blain Fineness: 3200 cm^2^/g (range 3200–3700 cm^2^/g)				
	Chemical Analysis		Recommended Values		
	SiO_2_		~21.0%		
	Al_2_O_3_		~6.3%		
	Fe_2_O_3_		~3.0%		
	CaO		~66%		
	MgO		~1.0%		
	SO_3_		<3.2%		
	L.O.I.		~2.5% as little as possible		
	Free CaO		<1.5%		
	Mineralogical Composition		Recommended Values		
	C_3_S		40–70%		
	C_2_S		<20%		
	C_3_A		7–10%		
	C_4_AF		5–15%		
PVA-polyvinyl acetate	Polyvinyl Acetate				
	Fiber length		6 mm		
	Thickness		1.6–2.0 dtex		
	Dry tensile strength		11.5 cN/dtex		
	Dry E-modulus		275		
	Elongation at dry rupture		7.4%		
Kaolin	Specific weight 2.6 g/cm^3^ Weight density 0.35–0.45 g/cm^3^ Chemical Analysis: SiO_2_ 46–49% Al_2_O_3_ 35% Na_2_O 0.07% K_2_O 1.0% MgO 0.2% L.O.I. max. 10%				
Lime	CaCO_3_ Specific weight 2.73 g/cm^3^				

**Table 3:** Specification of tested fiber cement boards.

Board Denotation	Type of Board	Board Thickness [mm]	View of Board
A	Board without recycled cellulose fibers	8.0	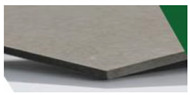
B	Board with 10% recycled cellulose fibers content	8.0	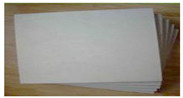
C	Board with 25% recycled cellulose fibers content	8.0	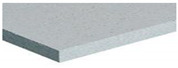
D	Board with 50% recycled cellulose fibers content	8.0	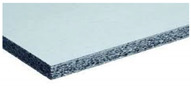

**Table 4:** Absorbability and moisture content of tested boards.

Board Denotation	Absorbability *n*_w_ [%] After Storage in Water for:							Moisture Content
	1 h	2 h	3 h	24 h	48 h	72 h	96 h	*w*_m_ [%]
A	6.40	7.15	7.73	8.02	10.88	11.90	11.91	1.18
B	9.14	11.08	11.72	14.22	19.05	20.51	16.51	7.62
C	11.24	11.89	12.34	16.78	21.45	22.20	22.21	10.15
D	12.89	13.74	14.54	18.38	22.86	23.64	23.65	10.87

**Table 5:** Bending test results for boards.

Board Denotation	Mean *MOR* [MPa] (In Two Directions) Acc. to [12] After Storage in Water for:							
	0 h (dry)	1 h	2 h	3 h	24 h	48 h	72 h	96 h
A	29.79	26.91	25.16	24.44	19.28	17.34	16.80	16.65
B	20.71	19.18	17.23	15.87	13.41	12.50	12.20	12.12
C	16.00	15.11	14.67	13.15	11.07	10.59	10.47	10.27
D	13.18	12.22	11.56	10.76	9.45	7.28	6.87	6.76

**Table 6:** Registered AE events recognized as accompanying rupture of fibers during three-point bending.

Board Denotation	Count of AE Events Recognized as Accompanying Rupture of Fibers			
	0 h (dry)	1 h	2 h	24 h
A	429	241	137	41
B	456	264	102	29
C	364	119	97	21
D	277	114	77	19

## References

[1] Schabowicz K., Gorzelańczyk T., Ranachowski Z., Schabowicz K. (2018). Fabrication of fibre cement boards. The Fabrication, Testing and Application of Fibre Cement Boards.

[2] Bentchikou M., Guidoum A., Scrivener K., Silhadi K., Hanini S. (2012). Effect of recycled cellulose fibres on the properties of lightweight cement composite matrix. Constr. Build. Mater..

[3] Savastano H., Warden P.G., Coutts R.S.P. (2005). Microstructure and mechanical properties of waste fibre–cement composites. Cem. Concr. Compos..

[4] Coutts R.S.P. (2005). A Review of Australian Research into Natural Fibre Cement Composites. Cem. Concr. Compos..

[5] Schabowicz K., Szymków M. (2016). Ventilated facades made of fibre-cement boards (in Polish). Mater. Budowlane.

[6] Schabowicz K., Jóźwiak-Niedźwiedzka D., Ranachowski Z., Kudela S., Dvorak T. (2018). Microstructural characterization of cellulose fibres in reinforced cement boards. Arch. Civ. Mech. Eng..

[7] Schabowicz K., Gorzelańczyk T., Szymków M. (2019). Identification of the degree of fibre-cement boards degradation under the influence of high temperature. Autom. Constr..

[8] Schabowicz K., Gorzelańczyk T., Szymków M. (2019). Identification of the degree of degradation of fibre-cement boards exposed to fire by means of the acoustic emission method and artificial neural networks. Materials.

[9] Gorzelańczyk T., Schabowicz K., Szymków M. (2016). Non-destructive testing of fibre-cement boards, using acoustic emission (in Polish). Przegląd Spaw..

[10] Gorzelańczyk T., Schabowicz K. (2015). Tests of fibre-cement boards containing recycled materials. Mater. Bud..

[11] Ardanuy M., Claramunt J., Toledo Filho R.D. (2015). Cellulosic Fibre Reinforced Cement-Based Composites: A Review of Recent Research. Constr. Build. Mater..

[12] (2018). EN 12467–Cellulose Fibre Cement Flat Sheets. Product Specification and Test Methods. https://standards.cen.eu/dyn/www/f?p=204:110:0::::FSP_PROJECT,FSP_ORG_ID:66671,6110&cs=1151E39EDCD9EF75E3C2D401EB5818ACD.

[13] Gorzelańczyk T., Schabowicz K. (2019). Effect of freeze–thaw cycling on the failure of fibre-cement boards, assessed using acoustic emission method and artificial neural network. Materials.

[14] Adamczak-Bugno A., Gorzelańczyk T., Krampikowska A., Szymków M. (2017). Non-destructive testing of the structure of fibre-cement materials by means of a scanning electron microscope. Bad. Nieniszcz. Diagn..

[15] Claramunt J., Ardanuy M., García-Hortal J.A. (2010). Effect of drying and rewetting cycles on the structure and physicochemical characteristics of softwood fibres for reinforcement of cementitious composites. Carbohydr. Polym..

[16] Mohr B.J., Nanko H., Kurtis K.E. (2005). Durability of kraft pulp fibre-cement composites to wet/dry cycling. Cem. Concr. Compos..

[17] Pizzol V.D., Mendes L.M., Savastano H., Frías M., Davila F.J., Cincotto M.A., John V.M., Tonoli G.H.D. (2014). Mineralogical and microstructural changes promoted by accelerated carbonation and ageing cycles of hybrid fibre–cement composites. Constr. Build. Mater..

[18] Adamczak-Bugno A., Świt G., Krampikowska A. (2018). Time-frequency analysis of acoustic emission signals generated by cement-fiber boards during bending test. MATEC Web Conf..

[19] Li Z., Zhou X., Bin S. (2004). Fibre-Cement extrudates with perlite subjected to high temperatures. J. Mater. Civ. Eng..

[20] Kaczmarek M., Piwakowski B., Drelich R. (2017). Noncontact Ultrasonic Nondestructive Techniques: State of the Art and Their Use in Civil Engineering. J. Infrastruct. Syst..

[21] Stark W., Karbhari V.M. (2013). Non-destructive evaluation (NDE) of composites: Using ultrasound to monitor the curing of composites. Non-destructive Evaluation (NDE) of Polymer Matrix Composites. Techniques and Applications.

[22] Dębowski T., Lewandowski M., Mackiewicz S., Ranachowski Z., Schabowicz K. (2016). Ultrasonic tests of fibre-cement boards (in Polish). Przegląd Spawalnictwa.

[23] Drelich R., Gorzelanczyk T., Pakuła M., Schabowicz K. (2015). Automated control of cellulose fibre cement boards with a non-contact ultrasound scanner. Autom. Constr..

[24] Schabowicz K., Gorzelańczyk T. (2016). A non-destructive methodology for the testing of fibre cement boards by means of a non-contact ultrasound scanner. Constr. Build. Mater..

[25] Chady T., Schabowicz K., Szymków M. (2018). Automated multisource electromagnetic inspection of fibre-cement boards. Autom. Constr..

[26] Chady T., Schabowicz K. (2016). Non-destructive testing of fibre-cement boards, using terahertz spectroscopy in time domain. Bad. Nieniszcz. Diagn..

[27] Ranachowski Z., Ranachowski P., Dębowski T., Gorzelańczyk T., Schabowicz K. (2019). Investigation of structural degradation of fibre cement boards due to thermal impact. Materials.

[28] Schabowicz K., Ranachowski Z., Jóźwiak-Niedźwiedzka D., Radzik Ł., Kudela S., Dvorak T. (2016). Application of X-ray microtomography to quality assessment of fibre cement boards. Constr. Build. Mater..

[29] Ranachowski Z., Schabowicz K. (2017). The contribution of fibre reinforcement system to the overall toughness of cellulose fibre concrete panels. Constr. Build. Mater..

[30] Ranachowski Z. (1996). The application of neural networks to classify the acoustic emission waveforms emitted by the concrete under thermal stress. Arch. Acoust..

[31] Ranachowski Z., Jóźwiak-Niedźwiedzka D., Brandt A.M., Dębowski T. (2012). Application of acoustic emission method to determine critical stress in fibre reinforced mortar beams. Arch. Acoust..

[32] Yuki H., Homma K. (1996). Estimation of acoustic emission source waveform of fracture using a neural network. NDT E Int..

[33] Schabowicz K. (2005). Neural networks in the NDT identification of the strength of concrete. Arch. Civ. Mech. Eng..

[34] Łazarska M., Woźniak T., Ranachowski Z., Trafarski A., Domek G. (2017). Analysis of acoustic emission signals at austempering of steels using neural networks. Met. Mater. Int..

[35] Woźniak T.Z., Ranachowski Z., Ranachowski P., Ozgowicz W., Trafarski A. (2014). The application of neural networks for studying phase transformation by the method of acoustic emission in bearing steel. Arch. Civ. Mech. Eng..

[36] Rucka M., Wilde K. (2013). Experimental study on ultrasonic monitoring of splitting failure in reinforced concrete. J. Nondestr. Eval..

[37] Rucka M., Wilde K. (2015). Ultrasound monitoring for evaluation of damage in reinforced concrete. Bull. Pol. Acad. Sci.-Tech..

[38] Schabowicz K., Ventilated F. Fibre-Cement Board Production Technology and Testing Methods. http://www.oficyna.pwr.edu.pl/ksiazki/elewacje-wentylowane-technologia-produkcji-i-metody-badania-plyt-wloknisto-cementowych/.

